# Seroprevalence of *Helicobacter pylori* infection among children of low socioeconomic level in São Paulo

**DOI:** 10.1590/S1516-31802010000400002

**Published:** 2010-07-01

**Authors:** Aurea Cristina Portorreal Miranda, Rodrigo Strehl Machado, Edina Mariko Koga da Silva, Elisabete Kawakami

**Affiliations:** I PhD. Postgraduate student, Department of Pediatrics, Division of Pediatric Gastroenterology, Universidade Federal de São Paulo — Escola Paulista de Medicina (Unifesp-EPM), São Paulo, Brazil.; II PhD. Attending physician, Department of Pediatrics, Division of Pediatric Gastroenterology, Universidade Federal de São Paulo — Escola Paulista de Medicina (Unifesp-EPM), São Paulo, Brazil.; III PhD. Professor, Department of Pediatrics, Universidade Federal de São Paulo — Escola Paulista de Medicina (Unifesp-EPM), São Paulo, Brazil.; IV PhD. Professor, Department of Pediatrics, Division of Pediatric Gastroenterology, Universidade Federal de São Paulo — Escola Paulista de Medicina (Unifesp-EPM), São Paulo, Brazil.

**Keywords:** Helicobacter infections, Serology, Cross-sectional studies, Seroepidemiologic studies, Urban population, Infecções por helicobacter, Sorologia, Estudos transversais, Estudos soroepidemiológicos, População urbana

## Abstract

**CONTEXT AND OBJECTIVE:**

*Helicobacter pylori* infection is mainly acquired during childhood, and is associated with significant morbidity in adults. The aim here was to evaluate the seroprevalence and risk factors of *H. pylori* infection among children of low socioeconomic level attended at a public hospital in São Paulo, Brazil.

**DESIGN AND SETTING:**

Cross-sectional study, among patients attended at an outpatient clinic.

**METHODS:**

326 children were evaluated (150 boys and 176 girls; mean age 6.82 ± 4.07 years) in a cross-sectional study. Patients with chronic diseases or previous *H. pylori* treatment, and those whose participation was not permitted by the adult responsible for the child, were excluded. The adults answered a demographic questionnaire and blood samples were collected. The serological test used was Cobas Core II, a second-generation test. Titers > 5 U/ml were considered positive.

**RESULTS:**

*H. pylori* infection was diagnosed in 116 children (35.6%). Infected children were older than uninfected children (7.77 ± 4.08 years versus 5.59 ± 3.86 years; p < 0.0001). The seroprevalence increased from 20.8% among children aged two to four years, to 58.3% among those older than 12 years. There were no significant relationships between seropositivity and gender, color, breastfeeding, number of people in the home, number of rooms, bed sharing, living in a shantytown, maternal educational level, family income or nutritional status. In multivariate analysis, the only variable significantly associated with *H. pylori* seropositivity was age.

**CONCLUSION:**

Infection had intermediate prevalence in the study population, and age was associated with higher prevalence.

## RESUMO

**CONTEXTO E OBJETIVO:**

A infecção por *Helicobacter pylori* é adquirida principalmente na infância e é associada a morbidade significativa em adultos. O objetivo foi avaliar a soroprevalência de infecção por *H. pylori* bem como fatores de risco em crianças de baixo nível socioeconômico atendidas em um hospital público em São Paulo, Brasil.

**TIPO DE ESTUDO E LOCAL:**

Estudo transversal, realizado entre pacientes atendidos em ambulatório.

**MÉTODOS:**

Foram avaliadas 326 crianças (150 meninos e 176 meninas, idade: média 6,82 ± 4,07 anos) em estudo transversal. Foram excluídas aquelas com doenças crônicas, tratamento prévio de *H. pylori* e cujo responsável não consentiu participar do estudo. Os responsáveis responderam questionário demográfico e foi coletada uma amostra de sangue. O teste sorológico empregado foi o CobasCore II, exame de segunda geração, sendo considerados positivos títulos > 5 U/ml.

**RESULTADOS:**

Infecção por *H. pylori* foi diagnosticada em 116 (35,6%). A idade dos pacientes infectados foi maior que a dos demais (7,77 ± 4,08 anos *versus* 5,59 ± 3,86 anos; p < 0,0001). A prevalência aumenta de 20,8% entre dois e quatro anos de idade para 58,3% entre maiores de 12 anos. Não houve relação significativa entre soropositividade e sexo, raça, aleitamento materno, número de pessoas ou de cômodos na casa, compartilhamento de camas, domicílio em favela, escolaridade materna, renda familiar ou estado nutricional. Na análise multivariada, a única variável significativamente associada a soropositividade foi idade.

**CONCLUSÃO:**

A infecção possui prevalência intermediária na população estudada, e a idade foi associada a maior prevalência.

## INTRODUCTION

*Helicobacter pylori* gastritis is associated with peptic disease, gastric mucosa-associated lymphoid tissue (MALT) lymphoma and gastric cancer.^1^ This infection is more prevalent in developing countries, and it is associated with poverty and social deprivation. Its main risk factors include overcrowded households, poor sanitation and poor water supply.^2^ *H. pylori-*related diseases are rare during childhood. However, this period of life is very important in the natural history of the infection, because this age group shows higher incidence of infection.^3^ Spontaneous elimination of the infection is rare, and the infection remains active indefinitely unless it is treated with antimicrobials.

Decreasing prevalence of this infection was observed during the twentieth century in developed countries, and this decrease was accompanied by a corresponding decrease in the incidence of gastric cancer and peptic ulcer disease.^4^ Increasing focus is being placed on developing strategies to reduce the prevalence of the infection in developing countries, because gastric cancer is even more prevalent in these countries.^5^ In order to accomplish this goal, it is important to have greater know-ledge of the epidemiology of this infection during childhood.

The prevalence of this infection is not uniform between societies, and it varies both geographically and according to social and ethnic group. In developed countries, the prevalence ranges from 11% to 32% in adults,^6,7^ and 10% to 16.7% in children^8,9^ However, some high risk groups exist. Recently, a nationwide American study revealed higher prevalence in the Hispanic population (61.6%) than in the Caucasian population (26.2%).^6^ The prevalence in developing countries ranges from 49.1% to 87% in adults,^10,11^ but it is decreasing in some of these countries. In South Korea, a recent study showed that there had been a significant decrease over the seven-year period from 1998 (66.9%) to 2005 (59.6%).^12^ The prevalence ranges from 9% to 78.6% among schoolchildren in developing countries,^13,14^ and in Brazil it ranges from 2.4% to 66.5%.^15,16^ Infection prevalence is higher in socioeconomically deprived communities and lower among individuals of Japanese descent.^15^

## OBJECTIVE

The aim of this study was to evaluate the *H. pylori* seroprevalence among children attended at a public hospital in São Paulo, Brazil.

## METHODS

A total of 326 children and adolescents (mean age: 6.82 ± 4.07 years; median 6.58 years; interquartile range: 3.42 to 10.17) were included over a three-month period. Children and adolescents were eligible for inclusion if they were registered at the outpatient service of Hospital São Paulo with a diagnosis of upper airway infection on weekdays during the daytime shifts. All eligible individuals were invited to participate, without any sampling procedure. The daytime shifts were selected for convenience. The exclusion criteria included chronic digestive diseases (including upper gastrointestinal symptoms) and previous treatment for *H. pylori* infection. Hospital São Paulo is a public hospital that attends socioeconomically deprived people from the south side of the city of São Paulo. This city is a multicultural metropolis whose population is comprised of people from several parts of the country and even from different countries.

All eligible children who were present at the hospital on three days per week were consecutively included in the study. After a child had been invited to participate in the study, the accompanying adult responsible for the child filled out a questionnaire that sought demographic data and information on the child's breastfeeding, the number of people living in the house, the number of rooms in the house, bed sharing, characteristics of the dwelling, the parents’ schooling levels and income levels. A blood sample (2 ml) was then collected and centrifuged, and the serum was stored at −20° C. The study was evaluated and approved by the Ethics Committee of the Universidade Federal de São Paulo. Participation was voluntary, and all the accompanying adults responsible for the children read and signed a consent form.

### Serological tests

Serological tests were performed using Cobas Core II (Roche Diagnostics Systems, Basel, Switzerland), which is a second-generation enzyme-linked immunoassay (ELISA) for detecting antibodies that are specific to *H. pylori*. This assay is highly immunogenic and contains purified antigens (urease and a 54 kD heat shock protein). These antigens are highly specific for *H. pylori,* and they do not cross-react with other bacteria.^17^ The assay was performed in accordance with the manufacturer's recommendations, in the laboratory of Hospital das Clínicas, Faculdade de Medicina da Universidade de São Paulo. Serum samples and positive and negative controls were diluted and incubated with antigen-coated pearls to allow binding of specific antibodies to the pearl. The pearls were then washed and incubated with the conjugate (peroxidase-sheep antibody anti-human immunoglobulin G, IgG). After washing again, the pearls were incubated with the substrate (tetramethylbenzidine and hydrogen peroxide). The color of the pearls changed to blue, because they were coated with a specific antibody against *H. pylori*. Finally, the reaction was then stopped with acid and the optical density was measured using a spectrophotometer at 450 nm. We took the cutoff to be 5 U/ml, which is in accordance with a previous study in which this cutoff provided 96.8% and 71.4% sensitivity and 61.9% and 70.2% specificity among teenagers and children, respectively.^18^

### Statistics

The continuous and categorized variables were described using the mean and standard deviation or median and interquartile range, as needed. Qualitative variables were described using proportions. The significance of associations between independent variables and the dependent variable (seropositivity) was investigated using Pearson's chi-square test and Student's t test. All variables that were associated with seropositivity at a significance level lower than 0.2 were included in a logistic regression model.^19^ P-values lower than 0.05 were considered to be significant. All the statistical tests were performed using the Stata 9.2 software for Windows (Stata Corp, College Station, USA).

## RESULTS

Our study revealed that 116 of the 326 children (35.6%) were seropositive for *H. pylori*. The ELISA readings among the seronegative children ranged from 0 to 4.92 (mean 1.0 ± 1.21), whereas they ranged from 5.27 to 997.79 (mean 80.18 ± 151.55) among the seropositive children.

**Figure 1:**
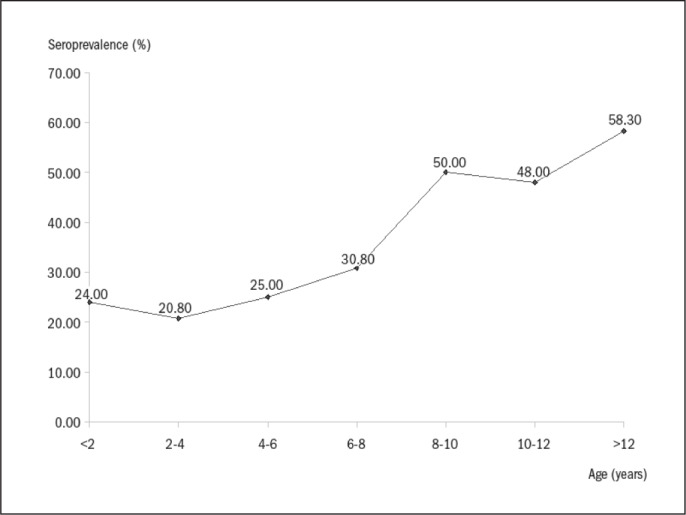
Seroprevalence of *Helicobacter pylori* infection according to age group among 326 children.

The infected children's ages were significantly higher than those of the non-infected children (7.77 ± 4.08 versus 5.59 ± 3.86; t = −4.78; P < 0.0001). Univariate analysis revealed a significant direct relationship between age and seropositivity [odds ratio, OR: 1.15 (95% confidence interval, CI: 1.08 – 1.22): P < 0.0001]. The increase in seroprevalence was steepest at the age of eight years, at which time it changed from 30.8% (between the ages of six and eight years) to 50% (ages eight to ten years) (Figure 1). The seroprevalence figures stabilized after this age and reached a level of 58.3% for teenagers (ages 12 to 18).

The other variables evaluated in the univariate analysis are shown in Table 1. The characteristics of the sewage collection system are not included because all households except seven (2.1% three of them seropositive) had access to the public sewer system. Only four mothers had received education at university level, and they were excluded from the analysis. In the multivariate analysis, adding the “number of rooms” and “number of people in the household” variables did not significantly change the logistic regression model (P = 0.6148). The only remaining variable that was significantly associated with seropositivity was age, and this had an adjusted odds ratio of 1.14 (95% CI: 1.07 – 1.21; P < 0.0001).

**Table 1.** Risk factors for *Helicobacter pylori* seroprevalence in univariate analysis among 326 children

Variable	*H. pylori* + n (%)	OR*	95% CI	P-value
Sex				
Female	62 (35.2)	1		
Male	54 (36.0)	1.03	0.66-1.63	0.88
Race				
White	9 (28.1)	1		
Nonwhite	107 (36.4)	1.46	0.65-3.28	0.35
Breast feeding				
No	59 (37.8)	1		
Exclusive until 4 months of age	57 (33.5)	0.83	0.53-1.31	0.42
Number of people per family household				
≤ 2	64 (39.3)	1		
> 2	52 (31.9)	0.72	0.46-1.14	0.17
Number of rooms per household		1.24	0.95-1.61	0.11
Bed sharing				
No	71 (33.8)	1		
Yes	45 (38.8)	1.24	0.77-1.99	0.37
Household in urban slum				
No	61 (38.6)	1		
Yes	55 (32.7)	0.77	0.49-1.22	0.27
Family income				
≥ 1 minimum wage	54 (33.5)	1		
< 1 minimum wage	62 (37.5)	1.19	0.76-1.88	0.45
Maternal schooling				
Illiteracy	7 (50)	1		
Less than 8 school years	90 (35)	0.54	0.18-1.58	0.26
Less than 11 school years	3 (30)	0.30	0.06-1.58	0.16
11 school years	15 (41.7)	0.71	0.21-2.47	0.59
University level	0 (0)		Not analyzed	
Nutritional status				
Adequate	63 (35.2)	1		
Malnourished†	53 (36.1)	1.03	0.66-1.64	0.87

* OR = odds ratio; CI = confidence interval;

† body mass index less than 10^th^ percentile.

## DISCUSSION

The seroprevalence in this study group was 35.6% in the study group, and this value was directly correlated with age. No other risk factors were found. The age-related increase in prevalence was most striking at the age of eight, at which time the prevalence changed from 30.8% (ages six to eight years) to 50% (ages eight to ten years). Another study in Brazil described similar results, including prevalence of 16.4% among infants, 36.7% among toddlers, and 62.1% among adolescents older than 15 years old.^20^ A recent study in a low-income community reported prevalence of 29% among infants and 59% among children older than six years of age.^21^ The increasing seroprevalence of *H. pylori* infection is a constant feature of its epidemiology, and the increase can be explained by two phenomena: constant incidence and a cohort effect.

Most adult patients acquire the infection during childhood, through various transmission pathways such as feco-oral, oro-oral or gastro-oral transmission.^3^ Intimate contact between an infected mother and her child provides a very important transmission route.^22^ The maximum incidence generally occurs before the age of four years in both developed and developing countries, and the incidence rates range from 2.1 to 11.7% and 14 to 26% in these countries, respectively.^23-26^ The incidence rate becomes lower in adulthood due to lower exposure risk and a reduction in the number of susceptible subjects.^24^ The cohort effect refers to the stable prevalence in adulthood that is established by the incidence rate during childhood.

The present study did not evaluate an adequate sample of the population, and it was restricted to a convenience sample of children attending a public hospital. This is an important limitation of the study. However, this flaw does not diminish the importance of the present study, because it provides data that are necessary for studying the epidemiology of *H. pylori* infection in other epidemiological studies. The decision to restrict the eligibility to children with respiratory symptoms avoided the selection bias inherent in infected individuals with digestive symptoms.^26,27^

The diagnostic tool for *H. pylori* infection in this study was a second-generation enzyme-linked immunoassay (Cobas Core II) that had previously been validated in Brazil. This method has shown 95.4% sensitivity and 100% specificity among adults,^28^ and the corresponding values are 86% and 71% among children.^18^ Its poorer accuracy among children is particularly observed among children younger than seven years of age: for these children the sensitivity is 44.4%.^29^ The main reason for the dissimilarity between children and adults involves the presence of different immunological host responses.^29^ The serological test detects specific IgG antibodies directed against bacterial antigens and it is unable to identify acute infection. ELISA kits for detecting specific immunoglobulin M (IgM) have poor accuracy, and they are not used within clinical routines, despite the recent finding that *H. pylori* infection elicits a host response involving the expression of specific IgM and IgG in the gastric mucosa.^30^ Other noninvasive diagnostic methods include the urea breath test and the *H. pylori* stool antigen test. These tests are more accurate during childhood. The urea breath test with ^13^C-urea (^13^C-UBT) uses a non-radioactive carbon isotope for detecting urease activity in the gastric mucosa. It identifies enrichment of ^13^CO_2_ in the isotope ratio of expirated air. ^13^C-UBT is highly sensitive (96.8%) and specific (93.2%), but it requires an expensive tracer and high-cost equipment (mass spectrometer or infrared spectrometer), which are not widely available.^31^ The stool antigen test is a direct enzyme immunoassay for detecting *H. pylori* antigens in stool samples. This test is highly sensitive (94.6%) and specific (96.5%), and it only requires equipment that is available in most laboratories. On the other hand, it needs to be locally validated.^32^ The stool antigen test had not been locally validated when the current study was performed. Serological tests are not useful in clinical settings because of their poor accuracy, but they are an appropriate method for use in epidemiological studies. They are widely used in this context because of their convenience and low cost. Additionally, this method can detect subjects that have been infected by the bacteria at any time during their lives, since the antibody titers remain positive even after eradication of the infection.

This study failed to identify any variable with a significantly association with infection, other than age. This lack of associations between infection and schooling level, race, income and overcrowding was surprising, because these variables were associated with the incidence of *H. pylori* infection in the univariate analysis in another study.^3^ Other variables, including the presence of an infected mother or infected sibling and nursing bottle usage were significantly associated with infection in a multivariate analysis.^3^ Another Brazilian study failed to identify any risk factors other than the presence of an infected mother, and this variable was not assessed in the current study.^21^ Infected relatives (especially the mother, but also siblings) are important in the epidemiology of the *H. pylori* infection.^22,25^ It is possible that other risk factors associated with infection are epiphenomena when this variable is not assessed. This could explain the wide range of habits and conditions, including cutlery sharing, bed sharing, overcrowding, consumption of raw food and presence of pets, that have been found to be associated with infection in different studies.^33-36^ A recent study on the seroprevalence of infection among blood donors identified race (nonwhite), low educational level, previous endoscopy and age as risk factors for *H. pylori* seropositivity in a multivariate analysis.^37^ Other authors have stressed that the water supply could be the source of the infection, but this variable was not tested in the present study, since all the subjects had access to the same water distribution system.^25,38^

## CONCLUSIONS

This study showed that *Helicobacter pylori* presented relatively high prevalence among the study population, and that age was associated with higher prevalence.

## References

[1] Malfertheiner P, Megraud F, O'Morain C (2007). Current concepts in the management of *Helicobacter pylori* infection: the Maastricht III Consensus Report. Gut.

[2] Windle HJ, Kelleher D, Crabtree JE (2007). Childhood *Helicobacter pylori* infection and growth impairment in developing countries: a vicious cycle?. Pediatrics.

[3] Rowland M, Daly L, Vaughan M (2006). Age-specific incidence of *Helicobacter pylori*. Gastroenterology.

[4] Rupnow MF, Shachter RD, Owens DK, Parsonnet J (2000). A dynamic transmission model for predicting trends in *Helicobacter pylori* and associated diseases in the United States. Emerg Infect Dis.

[5] Graham DY, Shiotani A (2005). The time to eradicate gastric cancer is now. Gut.

[6] Kruszon-Moran D, McQuillan GM (2005). Seroprevalence of six infectious diseases among adults in the United States by race/ethnicity: data from the third national health and nutrition examination survey, 1988--94. Adv Data.

[7] Thjodleifsson B, Asbjörnsdottir H, Sigurjonsdottir RB (2007). Seroprevalence of *Helicobacter pylori* and cagA antibodies in Iceland, Estonia and Sweden. Scand J Infect Dis.

[8] O'Donohoe JM, Sullivan PB, Scott R (1996). Recurrent abdominal pain and *Helicobacter pylori* in a community-based sample of London children. Acta Paediatr.

[9] Yamashita Y, Fujisawa T, Kimura A, Kato H (2001). Epidemiology of *Helicobacter pylori* infection in children: a serologic study of the Kyushu region in Japan. Pediatr Int.

[10] Olmos JA, Ríos H, Higa R (2000). Prevalence of *Helicobacter pylori* infection in Argentina: results of a nationwide epidemiologic study. Argentinean Hp Epidemiologic Study Group. J Clin Gastroenterol.

[11] Newton R, Ziegler JL, Casabonne D (2006). *Helicobacter pylori* and cancer among adults in Uganda. Infect Agent Cancer.

[12] Yim JY, Kim N, Choi SH (2007). Seroprevalence of *Helicobacter pylori* in South Korea. Helicobacter.

[13] Malaty HM, Kim JG, Kim SD, Graham DY (1996). Prevalence of *Helicobacter pylori* infection in Korean children: inverse relation to socioeconomic status despite a uniformly high prevalence in adults. Am J Epidemiol.

[14] Aguemon BD, Struelens MJ, Massougbodji A, Ouendo EM (2005). Prevalence and risk-factors for *Helicobacter pylori* infection in urban and rural Beninese populations. Clin Microbiol Infect.

[15] Ito LS, Oba-Shinjo SM, Shinjo SK (2006). Community-based familial study of *Helicobacter pylori* infection among healthy Japanese Brazilians. Gastric Cancer.

[16] Fialho AM, Braga AB, Queiroz DM (2007). The association between *Helicobacter pylori* infection and height in children from an urban community in north-east Brazil. Ann Trop Paediatr.

[17] Weiss J, Mecca J, da Silva E, Gassner D (1994). Comparison of PCR and other diagnostic techniques for detection of *Helicobacter pylori* infection in dyspeptic patients. J Clin Microbiol.

[18] Portorreal A, Kawakami E (2002). Avaliação do método imunoenzimático (ELISA) para diagnóstico da infecção por *Helicobacter pylori* em crianças e adolescentes [Evaluation of enzyme-linked immunosorbent assay for the diagnosis of *Helicobacter pylori* infection in children and adolescents]. Arq Gastroenterol.

[19] Vittinghof E, Glidden DV, Shiboski SC, McCulloch CE (2005). Regression methods in biostatistics: linear, logistic, survival, and repeated measures models (Statistics for Biology and Health).

[20] Oliveira AM, Queiroz DM, Rocha GA, Mendes EN (1994). Seroprevalence of *Helicobacter pylori* infection in children of low socioeconomic level in Belo Horizonte, Brazil. Am J Gastroenterol.

[21] Braga AB, Fialho AM, Rodrigues MN (2007). *Helicobacter pylori* colonization among children up to 6 years: results of a community-based study from Northeastern Brazil. J Trop Pediatr.

[22] Escobar ML, Kawakami E (2004). Evidence of mother-child transmission of *Helicobacter pylori* infection. Arq Gastroenterol.

[23] Granström M, Tindberg Y, Blennow M (1997). Seroepidemiology of *Helicobacter pylori* infection in a cohort of children monitored from 6 months to 11 years of age. J Clin Microbiol.

[24] Malaty HM, El-Kasabany A, Graham DY (2002). Age at acquisition of *Helicobacter pylori* infection: a follow-up study from infancy to adulthood. Lancet.

[25] Glynn MK, Friedman CR, Gold BD (2002). Seroincidence of *Helicobacter pylori* infection in a cohort of rural Bolivian children: acquisition and analysis of possible risk factors. Clin Infect Dis.

[26] Ozen A, Ertem D, Pehlivanoglu E (2006). Natural history and symptomatology of *Helicobacter pylori* in childhood and factors determining the epidemiology of infection. J Pediatr Gastroenterol Nutr.

[27] Opekun AR, Gilger MA, Denyes SM (2000). *Helicobacter pylori* infection in children of Texas. J Pediatr Gastroenterol Nutr.

[28] Rocha GA, Oliveira AM, Queiroz DM (1998). Serodiagnosis of *Helicobacter pylori* infection by Cobas Core ELISA in adults from Minas Gerais, Brazil. Braz J Med Biol Res.

[29] de Oliveira AM, Rocha GA, Queiroz DM (1999). Evaluation of enzyme-linked immunosorbent assay for the diagnosis of *Helicobacter pylori* infection in children from different age groups with and without duodenal ulcer. J Pediatr Gastroenterol Nutr.

[30] Sobala GM, Crabtree JE, Dixon MF (1991). Acute *Helicobacter pylori* infection: clinical features, local and systemic immune response, gastric mucosal histology, and gastric juice ascorbic acid concentrations. Gut.

[31] Kawakami E, Machado RS, Reber M, Patrício FR (2002). 13 C-urea breath test with infrared spectroscopy for diagnosing *Helicobacter pylori* infection in children and adolescents. J Pediatr Gastroenterol Nutr.

[32] Raguza D, Granato CF, Kawakami E (2005). Evaluation of the stool antigen test for *Helicobacter pylori* in children and adolescents. Dig Dis Sci.

[33] Elitsur Y, Yahav J (2005). *Helicobacter pylori* infection in pediatrics. Helicobacter.

[34] Hopkins RJ, Vial PA, Ferreccio C (1993). Seroprevalence of *Helicobacter pylori* in Chile: vegetables may serve as one route of transmission. J Infect Dis.

[35] Webb PM, Knight T, Greaves S (1994). Relation between infection with *Helicobacter pylori* and living conditions in childhood: evidence for person to person transmission in early life. BMJ.

[36] Kikuchi S, Ohgihara A, Hasegawa A (2004). Seroconversion and seroreversion of *Helicobacter pylori* antibodies over a 9-year period and related factors in Japanese adults. Helicobacter.

[37] Zaterka S, Eisig JN, Chinzon D, Rothstein W (2007). Factors related to *Helicobacter pylori* prevalence in an adult population in Brazil. Helicobacter.

[38] Klein PD, Graham DY, Gaillour A, Opekun AR, Smith EO (1991). Water source as risk factor for *Helicobacter pylori* infection in Peruvian children. Gastrointestinal Physiology Working Group. Lancet.

